# Preceding crops may reduce denitrification potential and enhance ammonium assimilation pathways

**DOI:** 10.3389/fmicb.2026.1808894

**Published:** 2026-04-22

**Authors:** Juan Li, Ming Liu, Chengwei Yang, Zhiyong Fan, Jiaen Su, Yanxia Hu, Yinju Yang, Junying Li, Yi Pu, Erdeng Ma, Xiaopeng Deng, Junwei Sun

**Affiliations:** 1 Dali Prefecture Branch of Yunnan Tobacco Company, Dali, Yunnan, China; 2 Yunnan Academy of Tobacco Agricultural Sciences, Kunming, China

**Keywords:** ammonium nitrogen, crop rotation, denitrification, metagenomics, tobacco

## Abstract

**Background:**

Soil microorganisms are pivotal to nitrogen (N) cycling in croplands, yet how preceding crops modulate their functional profiles remains unclear.

**Objective:**

This field study aimed to quantify the effects of barley (BT) and rapeseed (RT) preceding crops (vs. no preceding crop, CK) on soil microbial functions and N-metabolic pathways in tobacco fields.

**Results:**

High-throughput metagenomics revealed that BT and RT significantly increased soil microbial richness (Chao1 index) compared to CK. At the genus level, CK contained 64% and 24% fewer unique taxa than BT and RT, respectively. While the top five KEGG functional pathways (e.g., Metabolic pathways, Biosynthesis of secondary metabolites) were conserved across treatments, their relative abundances differed. Critically, preceding crops reduced soil denitrification rates and increased glutamine dehydrogenase activity. Redundancy analysis confirmed that ammonium-N concentration was the key edaphic factor strongly correlated with microbial community structure and function (*P* < 0.01).

**Conclusion:**

Our findings demonstrate that barley and rapeseed preceding crops enhance microbial richness and activity, thereby inhibiting denitrification and promoting N fixation via altered ammonium-N dynamics.

## 1 Background

In agricultural production, soil quality and health are fundamental to sustaining crop yields and farmland sustainability. With the global population increasing and agricultural intensification advancing, optimizing soil management to enhance soil fertility and ecological functions has become one of the key challenges faced by modern agriculture (Zhou et al., 2023; Al-Wabel et al., 2024; Duddigan et al., 2023). In this context, crop rotation systems have gained widespread attention due to their potential advantages in improving soil health, reducing pest and disease incidence, and increasing crop yields. Previous studies on crop rotation have been relatively comprehensive. For instance, Pierret demonstrated that deep-rooted crops (such as maize and alfalfa) can penetrate the soil profile, break up compacted layers, and increase soil porosity (Pierret, 2022). In contrast, shallow-rooted crops (such as wheat and soybeans) contribute to the aggregation and stabilization of surface soil through their dense root networks. However, the effects of different preceding crops on soil still require further research and exploration. The impact of different preceding crops on soil is significantly varied, particularly in terms of soil nutrient distribution, microbial community structure, and the diversity of microbial ecological functions (Götze et al., 2016; Blakney et al., 2023; Zhang S. et al., 2023). The key factors determining these differences include the root exudates, residue decomposition, and nutrient uptake and release patterns of the preceding crops. Leguminous crops, such as soybeans and alfalfa, have a unique nitrogen-fixing ability, allowing them to fix atmospheric nitrogen into the soil, significantly increasing the nitrogen content in the soil. This not only reduces the need for fertilizers for subsequent crops but also provides a rich nitrogen source for soil microbial communities, promoting the activity of nitrogen-cycling microorganisms (Sun et al., 2023; Ke et al., 2022). Additionally, the high content of proteins and other organic materials in leguminous crop residues provides an ideal substrate for the growth and reproduction of soil microorganisms, increasing the organic matter content in the soil and further improving soil fertility (Xie et al., 2022). In comparison, gramineous crops, such as maize and wheat, although lacking nitrogen-fixing abilities, contain residues rich in cellulose and lignin. These carbon sources decompose slowly in the soil, contributing to the formation of stable carbon pools. However, the high carbon-to-nitrogen ratio of gramineous crop residues may lead to short-term nitrogen leaching or fixation during decomposition, inhibiting the activity of certain nitrogen-cycling microorganisms (Xu et al., 2021).

In addition, root exudates are also a significant factor influencing soil microorganisms. Different crops secrete various substances through their roots, including organic acids, phenolic compounds, and polysaccharides. These exudates can serve directly as nutrients for microorganisms and also regulate soil physicochemical properties such as pH and redox potential, thereby affecting the structure and function of microbial communities (Panchal et al., 2022; Ma et al., 2022). The impact of preceding crops on soil microorganisms is multifaceted, involving changes in microbial community structure and the regulation of microbial functional diversity. By carefully selecting preceding crops, it is possible to optimize soil nutrient cycling and enhance soil fertility and crop productivity through the regulation of soil microbial community composition and function. This study focuses on the effects of different preceding crops on soil microbial community function and nitrogen cycling pathways. In practical agricultural production, balancing the nitrogen cycle is a critical goal for achieving efficient and sustainable agriculture. The impact of different preceding crops on soil nitrogen metabolism largely determines the nitrogen supply for subsequent crops and the overall nitrogen balance in the soil. The mechanisms by which preceding crops affect soil nitrogen metabolism are complex, involving multi-level and interwoven processes among soil, plants, and microorganisms. By selecting and managing preceding crops appropriately, these mechanisms can be regulated to optimize soil nitrogen cycling, improve nitrogen use efficiency in farmland, and ultimately enhance crop yields and maintain agricultural sustainability.

This study combines field experiments with metagenomic sequencing to clarify the mechanisms by which different preceding crops influence soil microbial community structure, functional potential, and nitrogen cycling processes in tobacco-planting soil. The primary objectives are to:

1. Quantify the legacy effects of preceding crops on soil microbial diversity, community composition, and functional gene profiles;
2. Elucidate the linkages among microbial community shifts, soil metabolite dynamics, and key nitrogen transformation pathways;
3. Evaluate the agronomic implications of preceding crop selection for improving soil fertility and nitrogen use efficiency.

Accordingly, the following hypotheses are proposed:

1. Different preceding crops exert distinct and persistent effects on the diversity, composition, and functional capacity of soil microbial communities in tobacco fields;
2. Crop-induced shifts in soil microbial communities mediate changes in soil metabolite profiles, which in turn regulate key processes of soil nitrogen cycling, including mineralization, nitrification, and denitrification.

Uncovering these mechanisms will provide a theoretical basis for optimizing cropping systems, enhancing soil health, and increasing nutrient utilization efficiency. This work also supports the development of sustainable agricultural management strategies for tobacco cultivation and similar rotational systems.

## 2 Materials and methods

### 2.1 Experimental site description and experimental design

This experiment was conducted in December 2023 in Dali City, Yunnan Province, China (100.30 °E, 25.23 °N, elevation 2,000 m). The soil type of the experimental site was typical red soil, and the main physical and chemical properties of the soil were as follows: bulk density 1.57 g·cm^−3^, pH 6.31, organic matter content 29.64 g·kg^−1^, total nitrogen content 1.16 g·kg^−1^, total phosphorus content 1.37 g·kg^−1^, total potassium content 35.94 g·kg^−1^, available phosphorus 19.04 mg·kg^−1^, available potassium 264.64 mg·kg^−1^, and alkaline hydrolyzable nitrogen 37.15 mg·kg^−1^.

The field experiment was designed using a randomized block design with three treatments, each with three replicates. Each plot was 10 m long and 10 m wide, covering an area of 100 m^2^. The previous crop in all plots was tobacco, of the variety Honghua Dajinyuan. The three treatments were as follows: (1) CK: no preceding crop; (2) BT: preceding crop was barley (variety: Kunlun No. 15); (3) RT: preceding crop was rapeseed (variety: Huayou No. 5). Both Kunlun No. 15 barley and Huayou No. 5 rapeseed are widely used local varieties. The barley and rapeseed were sown in December 2023. The row spacing for rapeseed was 25 cm, with a plant spacing of 20 cm, and for barley, the row spacing was 25 cm, with a plant spacing of 10 cm. Basal fertilizer, consisting of urea and compound fertilizer, was applied before planting. Urea was applied at a rate of 10 kg/mu, and compound fertilizer (15:15:15) at 12.5 kg/mu. Additional fertilization was carried out in February 2024, with urea applied at 2.5 kg/mu. All other field management practices followed local agronomic guidelines, and harvesting was conducted in May 2024.

### 2.2 . Soil sample collection and nitrogen structure determination

Soil samples were collected after crop harvest in 2024. For each treatment, 10 stubble residues were selected after harvest, Mix the 10 samples evenly and then divide them into 3 equal portions. The roots were excavated, and soil adhering to the root surfaces was gently shaken off. The remaining rhizosphere soil attached to the roots was gently brushed off and collected. A portion of this soil was immediately preserved in liquid nitrogen for soil metagenomic analysis. Another portion was air-dried in a shaded area for determining different forms of nitrogen in the soil. This study measured four nitrogen forms in the soil: total nitrogen (TN), soluble nitrogen (STN), ammonium nitrogen (AMN), and nitrate nitrogen (NIN). Additionally, four nitrogen forms were calculated: inorganic nitrogen (IN), organic nitrogen (ON), soluble organic nitrogen (SON), and insoluble organic nitrogen (ION). The Kjeldahl method with H_2_O_2_-H_2_SO_4_ was used for the determination of TN (LY/T1228-2015). The soil was treated with 1.8 mol/L NaOH solution, and the soil was hydrolyzed under alkaline conditions in a diffusion dish. After diffusion, the soil was absorbed by boric acid solution, and the content of STN was calculated by titration with standard acid (LY/T1228-2015). Fresh soil samples (10 g) were extracted with 50 mL of 2 M KCl, and AMN and NIN were measured using a continuous fow analyzer (SEAL AutoAnalyzer 3 HR, Germany; LY/T1228-2015). Inorganic nitrogen was calculated as ammonium nitrogen + nitrate nitrogen; organic nitrogen was calculated as total nitrogen—inorganic nitrogen; soluble organic nitrogen was calculated as soluble nitrogen—inorganic nitrogen; and insoluble organic nitrogen was calculated as organic nitrogen—soluble organic nitrogen.

### 2.3 Metagenomic analysis of rhizospheric microorganisms

#### 2.3.1 DNA extraction, detection and library construction

Total soil DNA from the rhizosphere soil samples was extracted from 500 mg of fresh samples using the PowerSoil^®^ DNA Isolation Kit (Qiagen, Hilden, Germany) following the manufacturer's protocols. After genomic DNA extraction, the quality of the extracted genomic DNA was assessed by 1% agarose gel electrophoresis. DNA was fragmented using the Covaris M220, and approximately 300 bp fragments were selected. The VAHTS Universal DNA Library Prep Kit for Illumina V3 (Illumina, America) was used for library construction. Sequencing was performed on the Illumina NovaSeq 6000 high-throughput sequencing platform, generating a large dataset with 141,412,057,534 high-quality reads. The average sequencing depth for each sample was approximately 7.86 Gb.

#### 2.3.2 Data quality control, preprocessing and assembly

The fastp v0.20.0 (https://github.com/OpenGene/fastp) was used to perform quality trimming at the 3′ and 5′ ends of the reads. The requirements were: (1) The number of unknown bases (N) should be less than 5; (2) Remove 50% of the base quality values less than 5 from the reads; (3) Remove the adapter sequences.

The Megahit v1.2.9 assembly software (https://github.com/voutcn/megahit) was used to assemble the optimized sequences, resulting in contigs for each sample.

#### 2.3.3 Gene prediction and abundance analysis

The contigs (with a length of ≥500 bp) assembled from the soil samples were subjected to ORF (Open Reading Frame Prediction) prediction using MetaGene Mark 3.38 (http://metagene.cb.k.u-tokyo.ac.jp/). Sequences with a length of ≥ 100 nt were selected. The ORF prediction results were de-redundant using CD-HIT 4.6.1 (http://www.bioinformatics.org/cd-hit/), and the non-redundant initial Unigenes were obtained. The Clean Data of each sample were compared with the non-redundant initial Unigenes using bwa 0.7.17, and the number of reads that the genes matched in each sample was calculated. The final Unigenes (with a read number > 2) were selected, and the abundance information of each gene in each sample was calculated and statistically analyzed.

#### 2.3.4 Species annotation

Using the software Kraken (version 2.1.2, http://ccb.jhu.edu/software/kraken), based on k-mers, the species classification of Unigenes was carried out through precise alignment. The Unigenes were compared with the database [sequences of bacteria (Bacteria), fungi (Fungi), and archaea (Archaea)], and also compared separately with each individual database. Through the annotation of bacteria, fungi and archaea, as well as the analysis of gene abundance, the data on the abundance and gene numbers of different treatments at various taxonomic levels (kingdom, phylum, class, order, family, genus, and species) were statistically compiled. Among them, the abundance calculation method for a certain species in each treatment is to sum up the abundances of all the genes annotated to this species; while the number of genes in each sample is obtained by counting the number of genes annotated to this species and with a non-zero abundance.

The Non-parametric factorial Kruskal-Wallis (KW) sum-rank test was used to detect features with significant abundance differences and to identify groups with significant differences in abundance. Metastats analysis and Lefse analysis were then conducted. Metastats analysis performed an inter-group *T*-test at each classification level, obtaining *p*-values, and then FDR corrected the *p*-values to obtain q-values. Lefse analysis used the Lefse1.0 software to estimate the magnitude of the effect of each component (species) abundance on the difference using linear discriminant analysis (LDA; the screening criterion was pvalue < 0.05 and LDAscore > 3).

#### 2.3.5 Microbial alpha diversity analysis

Based on the abundance tables at each classification level, the microbial Alpha diversity was obtained. Based on the abundance tables at each classification level, the microbial Alpha diversity was obtained. The Shannon index and Simpson diversity index were calculated, and the microbial community diversity in the samples was estimated. The Shannon index and Simpson diversity index were calculated, and the microbial community diversity in the samples was estimated.

### 2.4 Data analysis

The KEGG online service tool (http://www.genome.jp/kegg/) was used to annotate the proteins with KEGG. Subsequently, the differential proteins were enriched in the corresponding pathways using the KEGG mapper. The heat map was generated using the pheatmap package in R Studio (R 4.1.2) software, whereas the volcano plot and bubble diagram were generated using the ggplot2 package. Principal Component Analysis (PCA) was performed to ordinate the microbial composition in the different samples. The aggregated boosted tree analysis (ABT) was performed using the “gbm” package with 5,000 trees for boosting, 10-fold cross-validation, and three-way interactions. The data on endogenous hormones were analyzed using SPSS 22.0 (SPSS Institute Inc.) and Origin 8.0 (OriginLab). The data were analyzed using the online tools of the Majorbio Cloud Platform (https://cloud.majorbio.com/page/tools/). The sequence data is stored in the NCBI SRA database with the login number SUB14433325.

## 3 Results

### 3.1 Analysis of soil microbial diversity

The results of this study indicate that different preceding crops have varying effects on the diversity of soil microorganisms in tobacco-growing soils (Table 1). The Chao1 index in the CK treatment was significantly lower than in the other two treatments, being 2.28% and 2.48% lower than in the BT and RT treatments, respectively. Similarly, the Shannon index, Shannoneven index, and Simpsoneven index followed the same trend: the Shannon index in the CK treatment was 2.90% and 2.29% lower than in the BT and RT treatments, respectively; the Shannoneven index in the CK treatment was 2.72% and 1.98% lower than in the BT and RT treatments, respectively; and the Simpsoneven index in the CK treatment was 41.17% and 47.05% lower than in the BT and RT treatments, respectively. On the other hand, the Simpson index in the CK treatment was significantly higher than in the other two treatments, being 27.73% and 32.77% higher than in the BT and RT treatments, respectively.

**Table 1:** Analysis of α diversity of soil microorganisms under different treatments.

Sample	Chao1	Shannon	Simpson	Shannoneven	Simpsoneven
CK	24086.00 ± 265.93^b^	6.55 ± 0.02^b^	0.0119 ± 0.0050^b^	0.6496 ± 0.0019^b^	0.0034 ± 0.0001^b^
BT	24636.20 ± 414.37^a^	6.74 ± 0.10^a^	0.0086 ± 0.0014^a^	0.6673 ± 0.0110^a^	0.0048 ± 0.0009^a^
RT	24684.00 ± 446.46^a^	6.70 ± 0.30^a^	0.0080 ± 0.0078^a^	0.6625 ± 0.00273^a^	0.0050 ± 0.0005^a^

Different letters indicate significant differences between treatments, *p* < 0.05.

### 3.2 Analysis of soil microbial composition and functional pathways

**Figure 1:**
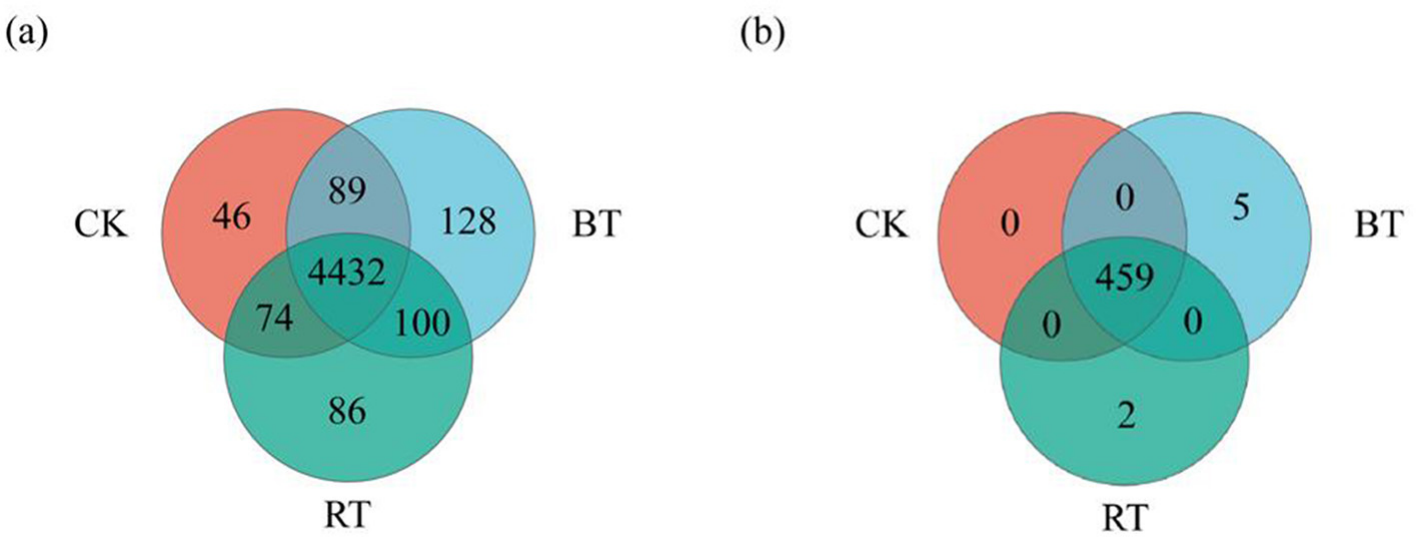
Venn diagram of soil microbial species composition and function under different treatments. **(a)** Venn diagram of soil microbial species composition in different treatments; **(b)** Venn diagram of soil functional composition under different treatments.

In this study, a Venn diagram analysis of soil microbial species at the genus level under different preceding crops was conducted (Figures 1a, b). The findings demonstrated that 4,641 microbial genera were detected under CK, 4,749 under the BT, and 4,692 under the RT. Among the three treatments, the BT treatment exhibited the highest microbial genus richness, whereas the CK showed the lowest. Additionally, 4,641 microbial genera were common across the CK, BT, and RT treatments. The CK treatment had 46 unique microbial species, which was significantly lower than the BT and RT treatments.

**Figure 2:**
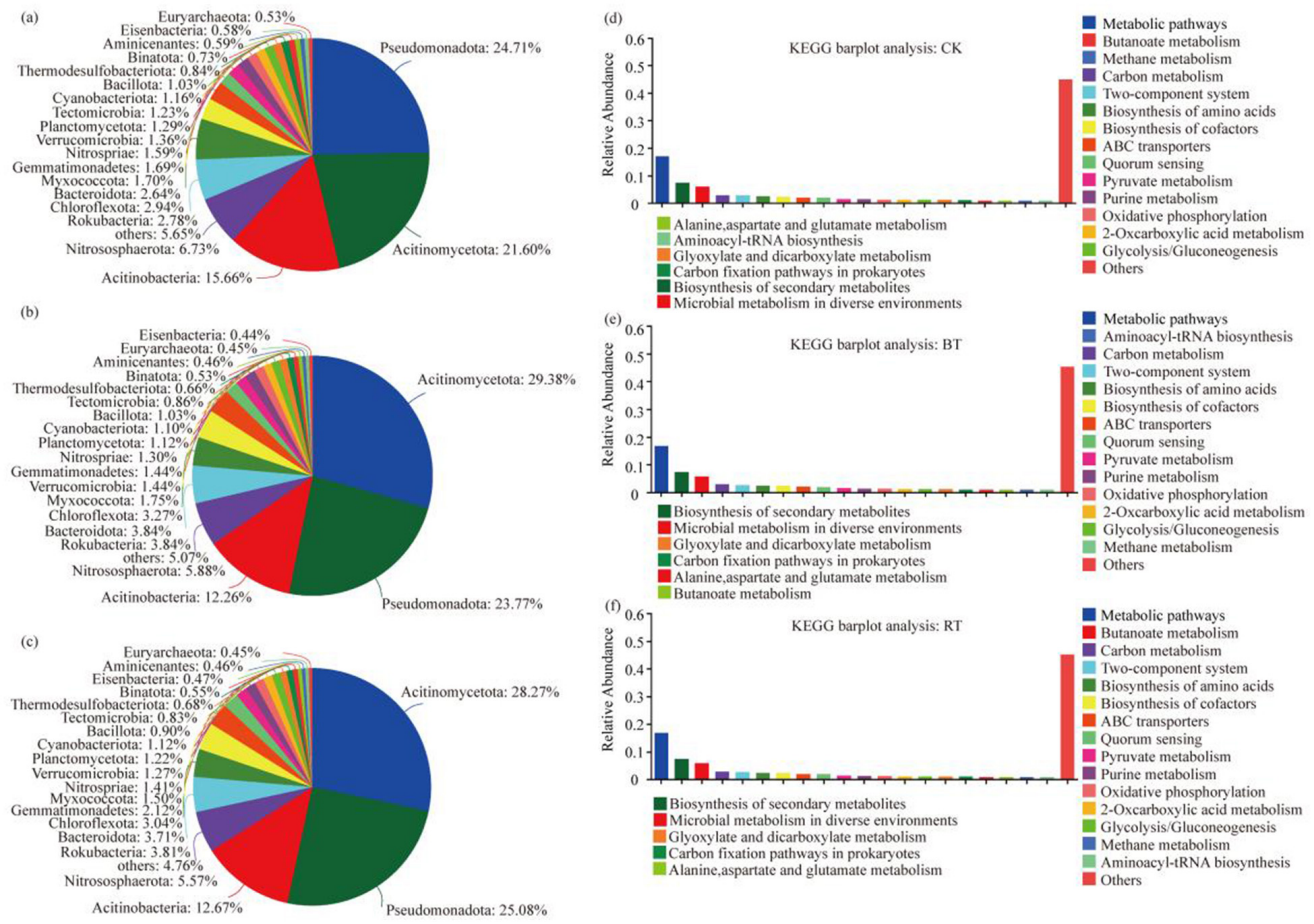
Pie chart of microbial species composition at each phylum level **(a)** CK; **(b)** BT; **(c)** RT. Bar diagram of Kegg functional composition of each treatment **(d)** CK; **(e)** BT; **(f)** RT.

**Figure 3:**
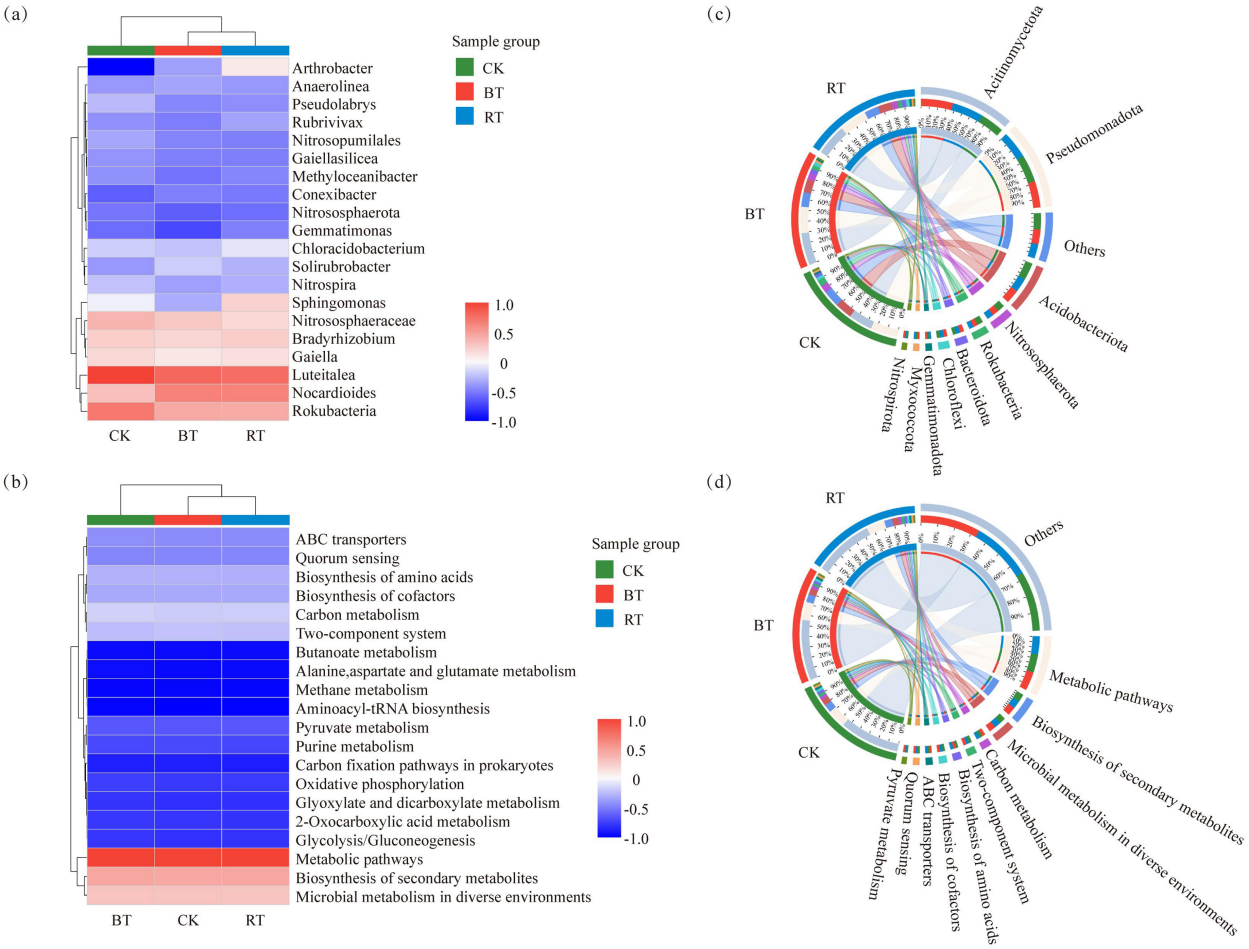
Heatmap of microbial community under different treatments **(a)**. KEGG function Heatmap **(b)**; microbial community Circos map under different treatments **(c)**; microbial community KEGG function Circos map under different treatments **(d)**.

When analyzing the proportion of relative abundance at the phylum level under different treatments (Figures 2a–c), it was found that the dominant phylum under the CK treatment was *Pseudomonadota*, accounting for 24.71% of all species in the CK treatment. In contrast, the dominant phylum under the BT and RT treatments was *Actinomycetota*, accounting for 29.38% and 28.27% of all species in the BT and RT treatments, respectively. However, the top four dominant phyla across the CK, BT, and RT treatments were consistently *Pseudomonadota, Actinomycetota, Acidobacteriota* and *Nitrospirota*. The analysis of KEGG functions under different treatments (Figures 2d–f) revealed that the top five KEGG functions across the CK, BT, and RT treatments were Metabolic pathways, Biosynthesis of secondary metabolites, Microbial metabolism in diverse environments, Carbon metabolism, and Two-component system, with differences only in the proportions of these functions. A correlation analysis of the treatments with microbial species composition and functional concentration (Figures 3a, b) showed significant differences in microbial species composition under different treatments. The dominant microbial species under the BT and RT treatments were more similar to each other and differed significantly from those under the CK treatment. However, the composition of KEGG functions was highly similar across treatments, with only minor differences. Furthermore, a co-occurrence analysis of microbial species (phylum level) and functions under different treatments (Figures 3c, d) revealed that *Actinomycetota* was the most dominant phylum across all three treatments, accounting for 27%, 37%, and 36% of all species in the CK, BT, and RT treatments, respectively. This was followed by *Pseudomonadota*, which accounted for 34%, 35%, and 34% of all species in the CK, BT, and RT treatments, respectively. In terms of KEGG functions, there were no significant differences in the concentration of functions across the three treatments, with only minor differences observed in specific functions. For example, the ratio of the Microbial metabolism in diverse treatment function accounted for 34% under the CK treatment and 33% under both the BT and RT treatments.

### 3.3 Analysis of differences in soil microbial communities and functional pathways

**Figure 4:**
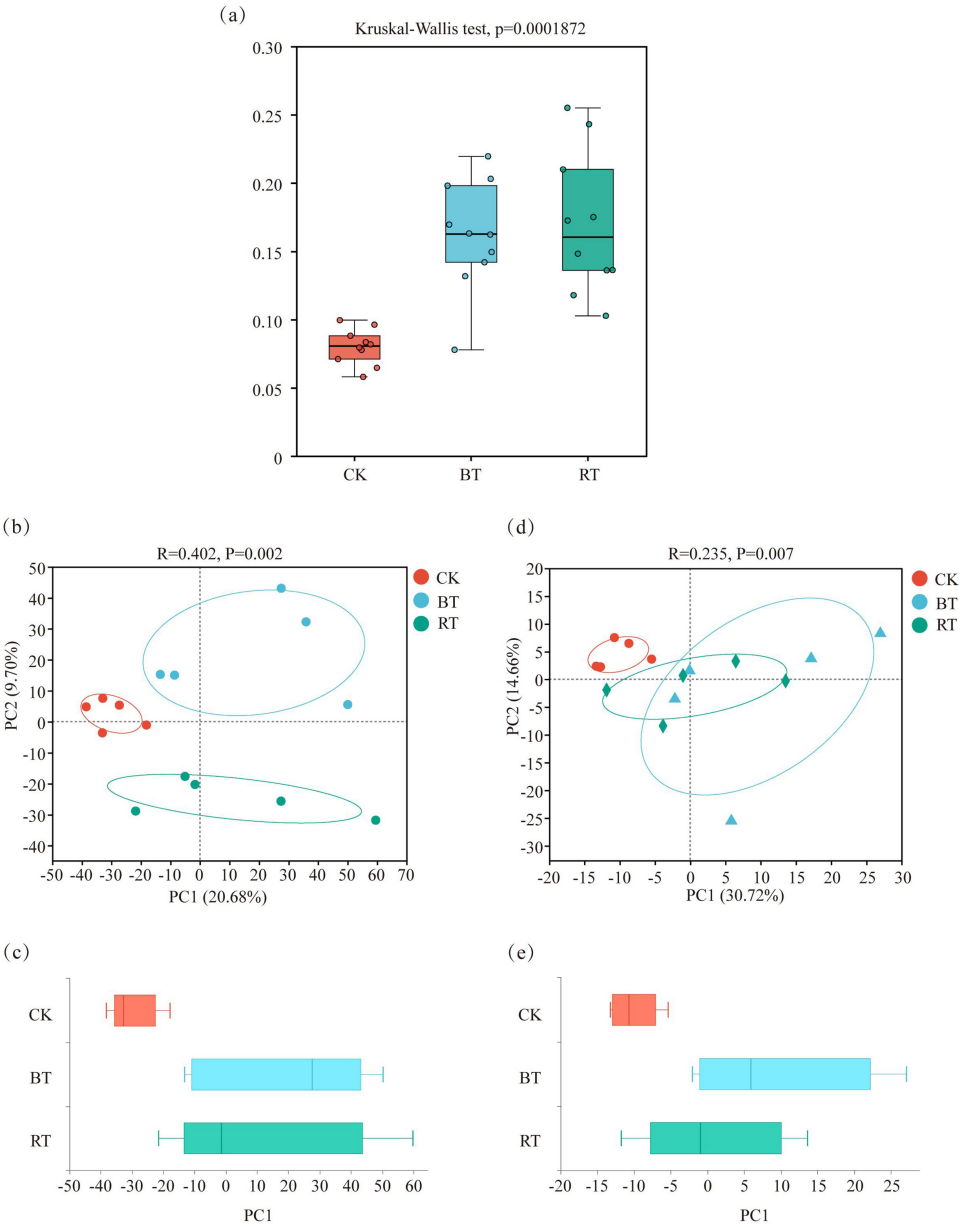
Beta diversity test of microbial communities under different treatments **(a)**. PCA analysis and box plot of microbial community under different treatments **(b**, **c)**; KEGG function analysis and box diagram of microorganisms under different treatments **(d**, **e)**.

This study analyzed the β-diversity of soil microbial communities in tobacco-growing soils under different preceding crops (Figure 4a). The results showed that the soil microbial community structures in the BT and RT treatments were relatively similar, while the microbial community structures in the CK treatment were more dispersed compared to BT and RT, indicating greater β-diversity distances. Further PCA analysis of soil microbial communities (Figures 4b, c) revealed that, although the microbial communities in the BT and RT treatments were highly similar, significant differences still existed between the treatments, as indicated by the non-overlapping 95% confidence ellipses. The boxplot results were consistent with the β-diversity boxplot results.

PCA analysis of soil microbial KEGG functions under different preceding crops (Figures 4d, e) showed that the similarity of soil microbial KEGG functions between the CK treatment and the BT and RT treatments was low, with significant differences observed, while the similarity of microbial KEGG functions between the BT and RT treatments was high.

**Figure 5:**
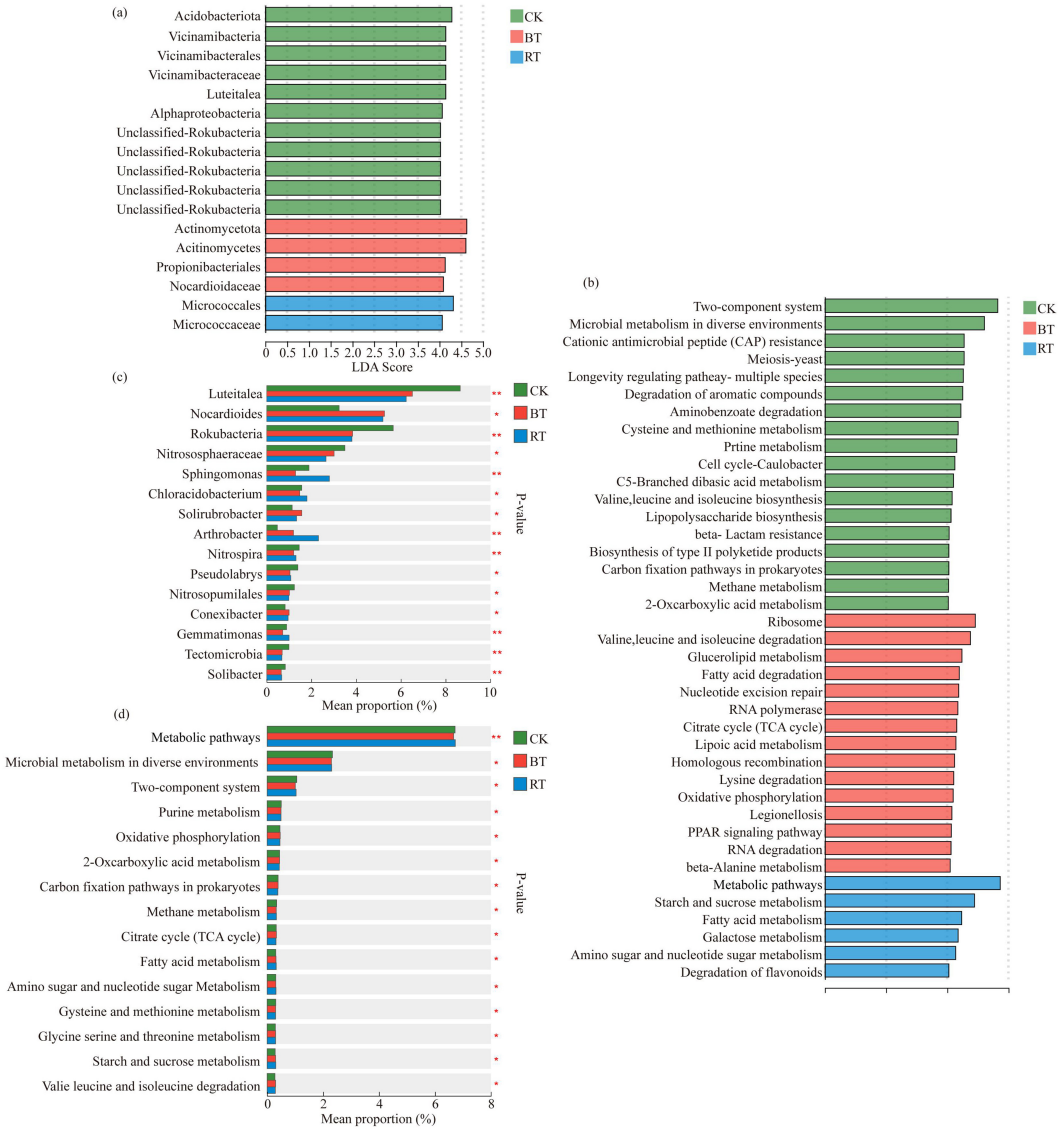
LDA discrimination results of microbial species difference test under different treatments, LDA threshold >4 **(a)**; LDA discrimination results were tested by the difference of microbial Kegg function under different treatments, LDA threshold >2 **(b)**; microbial species difference test under different treatments **(c)**. Analysis of differences in KEGG function of microorganisms under different treatments **(d)**. *means the differences between the treatments are significant (*p* < 0.05); **means the differences between the treatments are highly significant (*p* < 0.01); ***means the differences between the treatments are extremely significant (*p* < 0.001).

A linear discriminant analysis (LDA) was performed to identify microbial groups and functions with significant roles under different preceding crops. The results showed that the CK treatment had a higher number of significantly influential microbial groups; even when the LDA threshold was set to greater than 4, 11 species (ranging from phylum to genus level) were still identified. In contrast, the number of significantly influential microbial species in the BT and RT treatments at an LDA threshold >4 was 4 and 2, respectively (Figure 5a).

LDA analysis of microbial KEGG functions revealed that, when the LDA threshold was >2, there were 18 significantly influential microbial KEGG functions in the CK treatment, 15 in the BT treatment, and 6 in the RT treatment (Figure 5b). A multiple group comparison of microbial species among CK, BT, and RT treatments showed that 8 microbial genera exhibited highly significant differences (*P* ≤ 0.001) across treatments, including *Luteitalea, Rokubacteria, Sphingomonas, Arthrobacter, Nitrospira, Gemmatimonas, Tectomicrobia*, and *Solibacter*. Among these, *Luteitalea* and *Rokubacteria* had the highest percentages in the CK (Figure 5c). In the multiple group comparison of microbial KEGG functions among CK, BT, and RT treatments, only the Metabolic pathways function showed a highly significant difference (*P* ≤ 0.001) across the three treatments (Figure 5d). The percentage concentration of this function was highest in the RT treatment, followed by CK, and lowest in the BT treatment.

**Figure 6:**
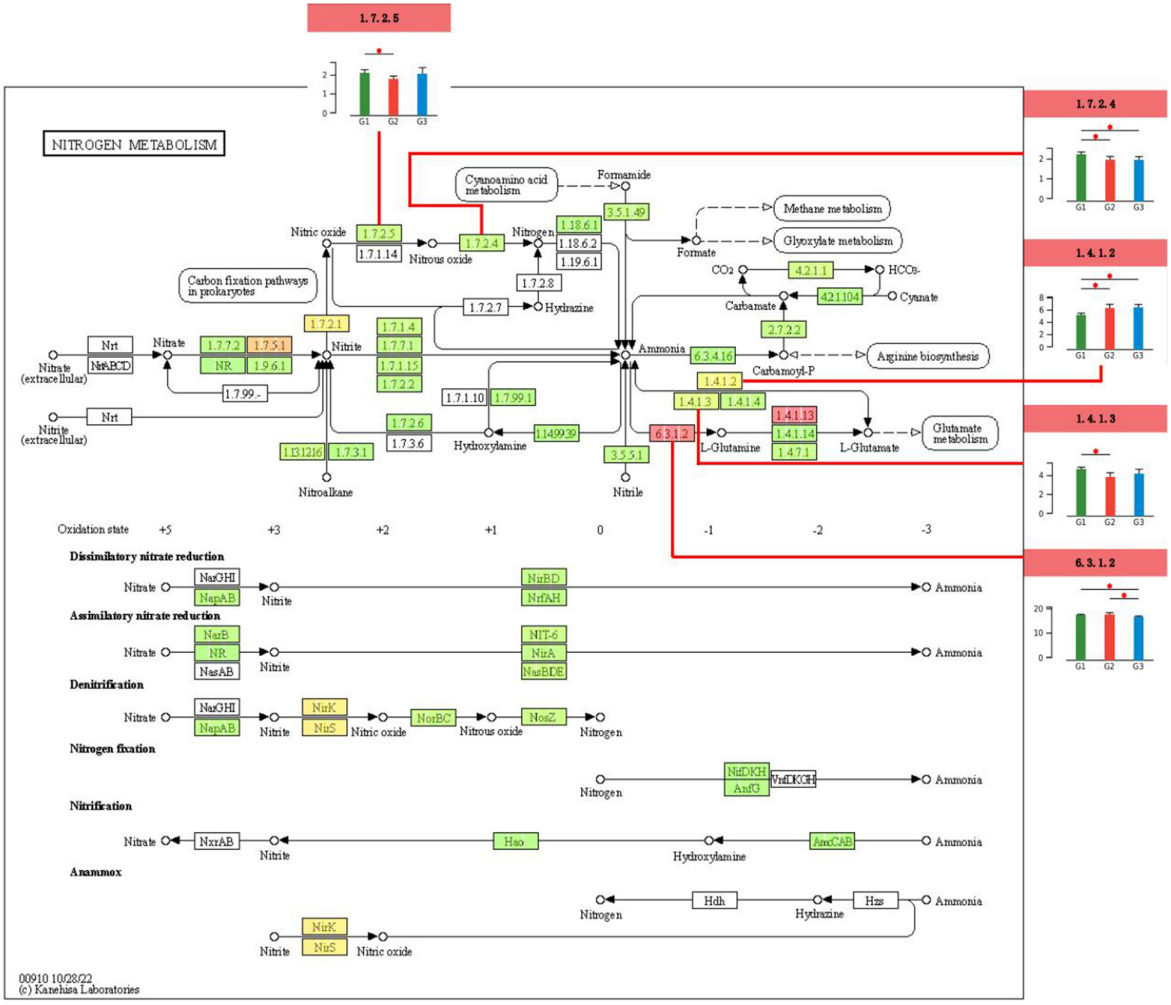
Differential test map of soil nitrogen metabolism pathways and differential analysis of key enzyme related gene concentration in different treatments. *means the differences between the treatments are significant (*p* < 0.05); **means the differences between the treatments are highly significant (*p* < 0.01); ***means the differences between the treatments are extremely significant (*p* < 0.001).

Based on KEGG annotation results, a differential concentration analysis and visualization of enzymes involved in the soil nitrogen metabolism pathway (Ko00910) were conducted. A pairwise significance analysis of the enzymes involved in the nitrogen metabolism pathway among CK, BT, and RT treatments was performed, with the results annotated for concentration and significance (Figure 6). The results indicated that the activities of soil nitrogen cycling-related enzymes differed significantly across the treatments, including nitric oxide reductase (NORs, 1.7.2.5), nitrous oxide reductase (N_2_ORs, 1.7.2.4), glutamate dehydrogenase (GDH, 1.4.1.2, 1.4.1.3), and glutamine synthetase (GS, 6.3.1.2). Notably, the activity of NORs was significantly higher in the CK treatment compared to the BT treatment, while the activities of N_2_ORs and GS were significantly higher in the CK treatment compared to both the BT and RT treatments. In contrast, the activity of GDH in the CK treatment was significantly higher than in the BT treatment, whereas the activity of GS in the BT and RT treatments were significantly higher than that in CK treatment.

### 3.4 Correlation analysis between soil nitrogen forms, microbial communities, and functional pathways

**Figure 7:**
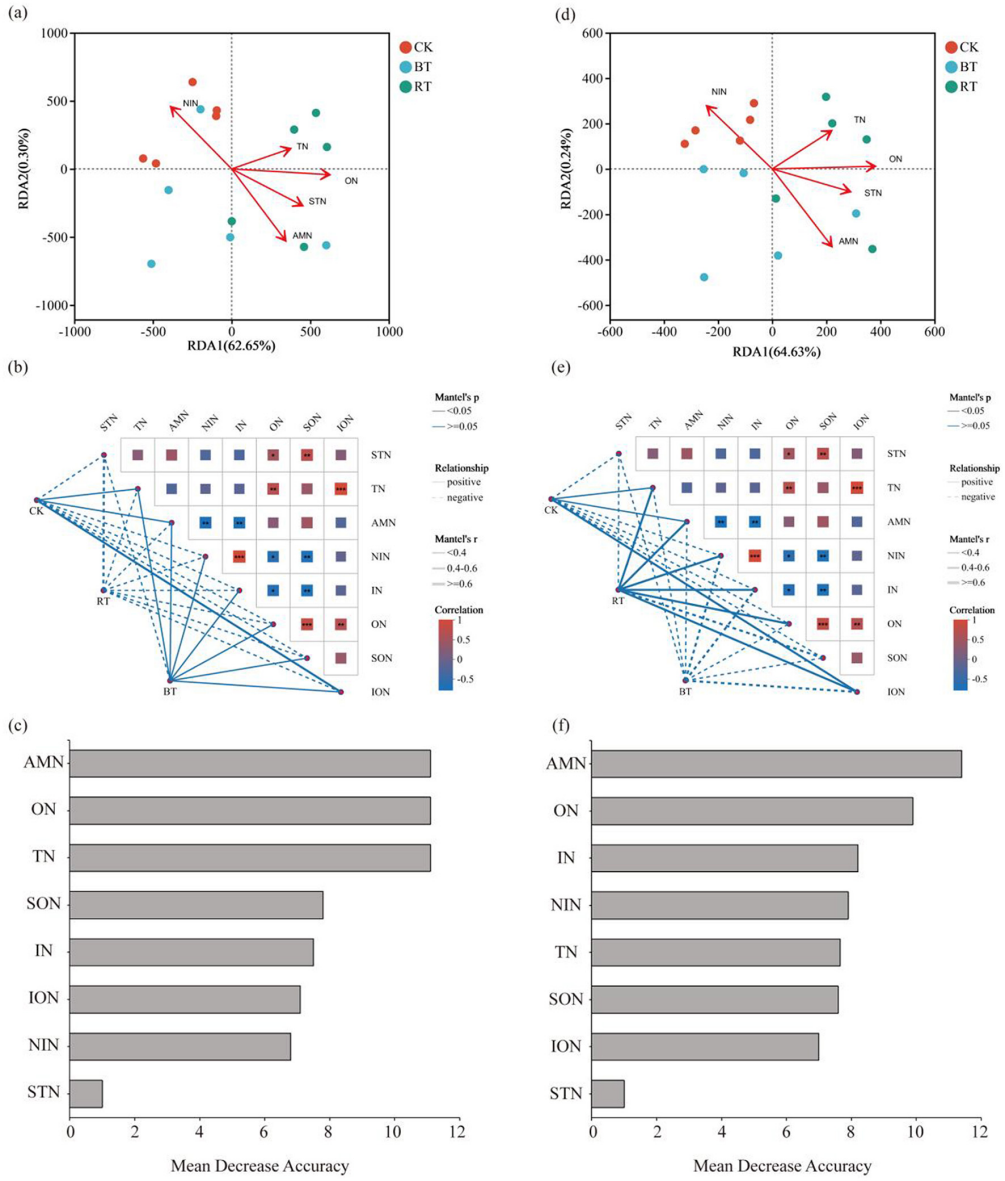
Correlation Analysis between soil nitrogen forms and microbial community composition under different treatments **(a–c)**; and correlation analysis between soil nitrogen forms and microbial KEGG function **(d–f)**.

A correlation analysis was conducted between environmental factors (soil nitrogen forms), microbial communities, and functional pathways (Figure 7). The results showed that microbial species under the CK treatment had a stronger correlation with nitrate nitrogen (NIN), while microbial species under the RT treatment were more strongly correlated with total nitrogen (TN), organic nitrogen (ON), soluble total nitrogen (STN), and ammonium nitrogen (AMN). Additionally, TN, ON, STN, and AMN exhibited a positive correlation with each other, while all these variables showed a negative correlation with NIN, with the strength of the correlation increasing.

Redundancy analysis (RDA) of soil nitrogen forms and microbial functional pathways yielded similar results to those of the microbial community analysis (Figures 7a, d). A Mantel test revealed that microbial species in the RT treatment were negatively correlated with all nitrogen structures, whereas microbial species in the CK treatment were negatively correlated with STN, NIN, inorganic nitrogen (IN), ON, and soluble organic nitrogen (SON), but positively correlated with TN, AMN, and insoluble organic nitrogen (ION). Microbial species in the BT treatment were positively correlated with NIN, IN, ON, SON, STN, NIN, IN, ON, and SON, but negatively correlated with STN (Figure 7b).

In the analysis of the relationship between soil nitrogen forms and microbial KEGG functions, it was found that microbial KEGG functions in the BT treatment were negatively correlated with all nitrogen structures, while those in the CK treatment were negatively correlated with STN, NIN, IN, and SON, but positively correlated with TN, AMN, and ION. In the RT treatment, microbial KEGG functions were negatively correlated with STN and SON, but positively correlated with TN, AMN, NIN, IN, and ION (Figure 7e).

The aggregated boosted tree (ABT) model was used to determine the relative influence of different nitrogen structures on microbial species and KEGG functions (Figures 7c, f). The results indicated that AMN, ON, and TN were the most important factors influencing microbial species, with variance coefficients of 11.8%, 11.7%, and 11.7%, respectively. AMN and ON were also the most influential factors affecting microbial KEGG functions, with variance coefficients of 11.7% and 10.4%, respectively.

## 4 Discussion

### 4.1 Preceding crops enriched the species and functional pathways of soil microbial communities in tobacco fields, with different effects depending on the crop

Continuous monocropping disrupts soil microecological balance and impairs soil health by altering microbial community structure and nutrient cycling efficiency. In contrast, crop rotation modulates the soil rhizosphere environment and microbial assemblages, which is a core strategy for maintaining agricultural sustainability. (Sanzovo et al. 2023) confirmed that crop rotation increased the abundance of slow-growing rhizobia in soil, effectively boosting soybean nitrogen acquisition and yield profitability. (Town et al. 2023) further demonstrated that rotation reshaped the composition of soil, rhizosphere and root microbial communities in rapeseed cultivation, with cascading effects on soil nutrient availability. In tobacco production systems, rotation has been widely verified to improve tobacco yield and quality, reduce soil-borne disease incidence, and restore soil health: (Wang et al. 2023) found that mushroom-tobacco rotation maintained the ecological balance of the soil microbial environment by regulating microbial community succession, while (Yan et al. 2024) reported that tobacco-maize rotation enhanced the stability and complexity of soil microbial networks, thus improving the potential ecological functions of tobacco-planting soil.

The results of this study further expand these findings, demonstrating that barley and rapeseed preceding crops significantly increase the α and β diversity of soil microbial species in tobacco fields, enhance microbial species richness and evenness, and optimize microbial community structure. Notably, although different preceding crops had little impact on the types of the top five KEGG functional pathways (Metabolic pathways, Biosynthesis of secondary metabolites, Microbial metabolism in diverse environments, Carbon metabolism, and Two-component system) across treatments, they altered the relative abundances of these pathways—this pattern is consistent with the findings of (Liu et al. 2024), who reported that preceding crops mainly affect the abundance rather than the composition of dominant soil microbial functional pathways, which is a typical manifestation of microbial functional redundancy in soil ecosystems. Microbial functional redundancy ensures the stability of core soil ecological processes (e.g., basic metabolism and nutrient cycling) under different agricultural management measures, and is a key mechanism for soil to resist external disturbances. While the core functional pathways remain conserved, the changes in their relative abundances reflect the fine-tuning of microbial metabolic activities in response to different preceding crop regimes, which is closely linked to the differences in soil nutrient supply and rhizosphere microenvironment induced by preceding crops.

The results of this study showed that barley and rapeseed preceding crops drive a shift in the dominant soil microbial phylum from *Pseudomonadota* to *Actinomycetota*, which is a critical mechanism for improving the health of tobacco-planting soil. (Lyng and Kovács 2023) noted that some members of *Pseudomonadota* have strong plant pathogenicity and can induce soil-borne diseases in continuous cropping systems, while although partial *Pseudomonadota* species contribute to soil bioremediation via polycyclic aromatic hydrocarbon degradation (Chen Z. et al., 2022), their dominant position in the no-preceding-crop treatment (CK) is associated with the disrupted soil microecology under continuous tobacco cropping. In contrast, *Actinomycetota (Actinomycetes)* have multiple beneficial ecological functions for soil and crops: (Bhatti et al. 2017) reported that *Actinomycetes* can inhibit the growth of rhizosphere plant pathogens through the secretion of antimicrobial secondary metabolites, decompose complex polymer mixtures (e.g., cellulose, lignin) from dead plants, animals and fungi via extracellular enzyme production, and directly participate in soil nitrogen fixation. The increased abundance of *Actinomycetota* in BT and RT treatments thus not only enhances soil disease suppression ability but also promotes nitrogen fixation and organic matter decomposition, laying a foundation for improved soil fertility and tobacco nitrogen acquisition.

The divergent effects of barley and rapeseed on soil microbial communities are fundamentally driven by differences in root exudates and C:N ratio of crop residues, as well as the unique metabolic characteristics of rapeseed. First, root exudates are the core signal and nutrient medium between plants and soil microorganisms: barley, as a gramineous crop, secretes root exudates dominated by carbohydrates and low-molecular-weight organic acids, while rapeseed releases exudates containing glucosinolates—a class of sulfur-containing secondary metabolites specific to Brassicaceae (Chen S. et al., 2022; Benitez et al., 2017). Glucosinolates and their hydrolysis products have selective antimicrobial effects, which can inhibit the growth of certain pathogenic microorganisms while promoting the proliferation of beneficial microbes such as *Micrococcales*, thus explaining the higher abundance of *Micrococcales* and *Micrococcaceae* in the RT treatment revealed by LDA analysis. In contrast, barley root exudates favor the enrichment of *Actinomycetota, Actinomycetes, Propionibacteriales*, and *Nocardioidaceae*, which are efficient in utilizing carbohydrate-rich substrates and participating in nitrogen cycling. Second, the C:N ratio differences between barley and rapeseed residues further regulate microbial community succession: barley residues have a higher C:N ratio (rich in cellulose and lignin), and their slow decomposition provides a continuous carbon source for slow-growing microbes such as *Actinomycetota*; rapeseed residues have a lower C:N ratio (rich in proteins and nitrogen-containing compounds), and their rapid decomposition releases available nitrogen, which promotes the proliferation of copiotrophic microbes such as *Micrococcales* and enhances the activity of nitrogen metabolism-related pathways.

In addition to the shifts in microbial taxa, the two preceding crops also induce differences in specific microbial functional pathways, with rapeseed significantly increasing the relative abundance of Tyrosine metabolism and Metabolic pathways compared to barley. The tyrosine metabolism pathway is the starting point for the synthesis of a variety of structurally diverse natural compounds in plants (Xu et al., 2019), and its enhanced activity in the RT treatment is closely linked to rapeseed glucosinolate metabolism and the associated microbial secondary metabolite synthesis. Tyrosine-derived metabolites (e.g., tocopherols, plastoquinone, ubiquinone) are essential for plant antioxidant defense and energy metabolism, and the increased abundance of this pathway in soil microbes indicates that rapeseed preceding crops can promote the synthesis of beneficial secondary metabolites in the rhizosphere, which further enhances the adaptability of tobacco to the soil environment. These results collectively confirm that preceding crops enrich the species and functional pathways of soil microbial communities in tobacco fields, and the crop-specific effects are the result of the combined action of root exudate characteristics, residue C:N ratio, and unique plant metabolic traits (e.g., rapeseed glucosinolates), with microbial functional redundancy maintaining the stability of core ecological processes while fine-tuning specific metabolic activities.

### 4.2 Preceding crops restricted the denitrification rate in soil nitrogen metabolism pathways and increased GDH activity

The nitrogen cycle is one of the most important biogeochemical cycles in the natural environment, with microbial activity being a key factor in controlling nitrogen transformation. Soil microorganisms are highly sensitive to environmental disturbances, and changes in the external environment can cause fluctuations in the population and quantity of soil microorganisms, further altering microbial diversity and community structure, which in turn affects the functioning of the nitrogen metabolism system in agricultural soils. The results of this study indicate that preceding crops enriched the species and functional pathways of soil microorganisms, with different effects depending on the crop. Changes in soil microorganisms can further influence the activity levels of soil nitrogen metabolism pathways (Zhao et al., 2024). Cui found that in a soybean continuous cropping system, the application of biochar altered the soil microbial community, changed rhizosphere metabolites, and subsequently affected nitrogen metabolism pathways (Cui et al., 2024). This study shows that during the soil nitrogen cycling process under different preceding crop treatments, the activities of soil nitrogen cycling-related enzymes exhibited significant differences across treatments, including NORs, N_2_ORs, GDH, and GS. The activities of NORs and NORs were significantly higher in soils without preceding crops compared to soils with preceding crops. Both enzymes are involved in the denitrification process, gradually reducing nitrate and nitrite, ultimately releasing nitrogen in the form of nitric oxide (NO), nitrous oxide (N_2_O), or molecular nitrogen (N_2_; Song et al., 2021). Kuypers indicated that NO is a signaling molecule and a toxic substance, while N_2_O is a potent greenhouse gas (310 times stronger than CO_2_ in terms of greenhouse effect and a major substance that depletes the ozone layer; Kuypers et al., 2018). The higher the activity of nitric oxide reductase and N_2_ORs, the more active the soil denitrification process. The results of this study indicate that planting preceding crops can slow down the reduction of nitric oxide and nitrous oxide in soil denitrification, reducing nitrogen loss from the soil. Moreover, among the preceding crops, rapeseed had a stronger inhibitory effect on the activities of NORs and N_2_ORs than barley. Glutamate dehydrogenase (GDH) is an enzyme found in soil that primarily catalyzes the oxidation of glutamate, converting it into alpha-ketoglutarate and ammonia (Huang et al., 2022; Zhang W. et al., 2023). As a key enzyme in the secondary pathway of ammonium assimilation, GDH plays a regulatory role in the entire process of ammonium assimilation and is involved in the synthesis and metabolism of glutamate, making it a critical enzyme in these processes (Rizwan et al., 2022). Some studies suggest that GDH may act as a compensatory mechanism when glutamine synthetase (GS) activity is reduced under adverse conditions, supporting the GS/GOGAT metabolic pathway (Song et al., 2022). The results of this study indicate that soils with preceding crops exhibited higher GDH activity, resulting in the production of more glutamine, which then participates in subsequent glutamate metabolism. Additionally, the effect of preceding crops on promoting GDH activity varied: rapeseed had a greater effect on promoting GDH activity than barley.

### 4.3 Preceding crops altered soil nitrogen forms, subsequently influencing soil microbial communities and functions

Soil nitrogen is one of the key factors influencing the structure and function of soil microbial communities. The proportion and availability of different nitrogen forms (such as ammonium nitrogen ${\text{NH}}_{4}^{-}$, nitrate nitrogen ${\text{NO}}_{3}^{-}$, and organic nitrogen) in the soil directly affect the living environment, metabolic activities, and community composition of microorganisms (Li et al., 2021). The results of this study indicate that nitrogen changes induced by preceding crops significantly affected the composition of soil microbial communities, with nitrogen availability being one of the critical driving factors for microbial growth. Microbial species composition in soils without preceding crops showed a strong correlation with nitrate nitrogen (NIN), whereas different preceding crops resulted in varying correlations between soil microbial species and different forms of nitrogen. Microbial communities in soils after rapeseed cultivation exhibited a stronger correlation with total nitrogen (TN) and organic nitrogen (ON). The study further reveals that AMN, ON, and TN all have significant impacts on microbial community composition, with AMN being particularly important among the nitrogen structures affecting microbial species. AMN typically promotes the proliferation of ammonia-oxidizing microorganisms (such as ammonia-oxidizing bacteria, AOB), which use ${\text{NH}}_{4}^{-}$ as an energy and electron donor, converting it into NIN (${\text{NO}}_{3}^{-}$) through ammonia oxidation (Fu et al., 2023; Dou et al., 2023). In ${\text{NH}}_{4}^{-}$-rich soils, these microorganisms often dominate. Conversely, in nitrate-rich soils, nitrifying and denitrifying bacteria are more active, as they can utilize ${\text{NO}}_{3}^{-}$ for energy metabolism and even reduce ${\text{NO}}_{3}^{-}$ to nitrogen gas (N_2_) or nitrous oxide (N_2_O) in anaerobic conditions, completing the denitrification process. The decomposition of ON (such as amino acids, proteins, etc.) relies on a series of heterotrophic microorganisms, which release inorganic nitrogen through the degradation of organic matter (Robinson et al., 2022). The activities of these heterotrophic microorganisms not only affect the mineralization rate of organic nitrogen but also indirectly shape the microbial community structure by altering soil pH and the availability of other nutrients through the production of organic acids as decomposition by-products. Changes in microbial community structure lead to changes in microbial functions. The study results indicate that denitrification was more intense in soils without preceding crops, resulting in nitrogen loss from the soil in the form of NO and N_2_O released into the atmosphere. Additionally, changes in nitrogen forms and concentrations can also impact the functional diversity of microbial communities. For example, in nitrogen-rich environments, the expression of nitrogen cycle-related functional genes (such as those involved in ammonia oxidation, nitrification, and denitrification) significantly increases. In low-nitrogen environments, however, microorganisms may exhibit more stress-resistant functions, such as oxidative stress responses and the synthesis of secondary metabolites (Li et al., 2022; Cheng et al., 2020). Certain specific microbial functions (such as cellulose degradation, carbon fixation, etc.) may be suppressed or enhanced by changes in soil nitrogen, ultimately affecting overall soil health and crop growth potential. The study results indicate that AMN remains an important environmental factor influencing soil microbial functions. Ammonium nitrogen serves as a crucial substrate for ammonia-oxidizing bacteria (AOB) and ammonia-oxidizing archaea (AOA). These microorganisms convert ${\text{NH}}_{4}^{-}$ into nitrogen oxides, such as nitrite (${\text{NO}}_{2}^{-}$), which is further converted into nitrate (${\text{NO}}_{3}^{-}$) through the process of nitrification. Therefore, the concentration of ammonium nitrogen directly determines the intensity and rate of nitrification. Wu demonstrated that nitrogen, specifically ammonium, significantly increased the microbial availability of PFASs (*p* < 0.05; Wu et al., 2023). The results of this study suggest that preceding crops not only altered soil microbial communities and functions but also that these changes in microbial communities and functions are strongly correlated with changes in soil nitrogen forms.

## 5 Conclusion

This study reveals that preceding crops significantly modify the composition and functional pathways of soil microbial communities in tobacco fields, with effects varying by crop type. Soils under preceding cropping were dominated by Actinomycetota, which enhanced soil nitrogen fixation. Rapeseed as a preceding crop notably strengthened microbial metabolic functions, while all preceding crops reduced soil denitrification and increased GDH activity. These effects were closely associated with changes in soil nitrogen forms, particularly ammonium nitrogen, which was strongly correlated with microbial community structure and function. These results highlight the value of preceding crops in regulating soil nitrogen cycling through microbial pathways, supporting sustainable nutrient management in tobacco cultivation. Limitations include the short experimental duration and limited crop types, which may restrict broader applicability. Future work should focus on long-term field trials across different regions and clarify the molecular mechanisms linking preceding crops, soil nitrogen dynamics, and key microbial taxa to optimize nitrogen-use efficiency in tobacco systems. However, we also believe that microbial communities often require multiple seasons or years to stabilize under crop rotation systems. Therefore, further research is needed to determine the long-term variation patterns of soil microorganisms.

## Data Availability

The original contributions presented in the study are publicly available. This data can be found here: https://ngdc.cncb.ac.cn/gsa/browse/CRA041250.
